# An economic evaluation of pre-discharge home assessment visits following hip fracture: Analysis from a randomised controlled trial

**DOI:** 10.1177/02692155241312065

**Published:** 2025-02-21

**Authors:** Kylee J. Lockwood, Nicholas F. Taylor, Katherine E. Harding, Natasha K. Brusco

**Affiliations:** 1 Occupational Therapy, School of Allied Health, Human Services & Sport, La Trobe University, Melbourne, Australia; 2 Physiotherapy, School of Allied Health, Human Services & Sport, La Trobe University, Melbourne, Australia; 3 Allied Health Clinical Research Office, Eastern Health, Melbourne, Australia; 4 Rehabilitation, Ageing and Independent Living (RAIL) Research Centre, School of Primary and Allied Health Care, Monash University, Frankston, Australia

**Keywords:** Occupational therapy, economic evaluation, older adults, home visit, re-admission

## Abstract

**Objective:**

To investigate cost-effectiveness of pre-discharge home assessment visits for patients recovering after hip fracture.

**Design:**

Cost-effectiveness analysis completed alongside a randomised controlled trial.

**Setting:**

Hospital wards and the community.

**Participants:**

Adults 50 years and over with hip fractures planning to return home.

**Intervention:**

Participants were randomised to either usual-care hospital-based discharge planning or usual-care hospital-based discharge planning with a pre-discharge home assessment visit by an occupational therapist.

**Main measures:**

Functional Independence Measure, EQ-5D-3L, and number of falls. Incremental cost-effectiveness ratios were calculated for changes at 30 days and 6 months.

**Results:**

Seventy-seven patients participated. Compared to usual care, the home assessment group likely had fewer falls at 30 days (incidence rate ratio = 0.41, 95% confidence interval (CI) 0.15 to 1.11) and increased functional independence at 6 months (11.2 units, 95% CI 4.2 to 18.2) but no difference in quality of life (0.0 units, 95% CI −0.1 to 0.1). The mean cost to provide a home assessment visit was A$135.70. A mean cost saving of A$6182 (95% CI −$6414 to $18,777) favoured the home assessment group. The incremental cost-effectiveness ratio found a saving of A$71,337 (95% CI −$998,930 to $411,409) in achieving clinically important changes in functional independence for the home assessment group at 6 months and a saving of A$34,832 (95% CI −$331,344 to $213,900) per fall avoided in favour of the home assessment group at 30 days.

**Conclusion:**

From a health service perspective, pre-discharge home assessments for patients after hip fracture are likely to be cost-effective in restoring functional independence and reducing falls.

## Introduction

A hip fracture is a serious injury associated with considerable health burden and an economic impact on the health care system. These injuries often require long periods of hospitalisation and rehabilitation and are often followed by permanent disability and ongoing restrictions on mobility and activities of daily living.^1–3^ The transition home after hospitalisation carries high risks, including increased chances of mortality, admission to residential care, further falls, and other adverse events.^1^

Health and social care costs following a hip fracture are substantial, but interventions that support patients during the transition home have the potential to help ease the burden on patients, families, and the healthcare system.^4,5^ A recent systematic review of 112 studies reported that total health and social care costs associated with providing care during the first 12 months after hip fracture were US$43,669.^6^ While inpatient care represented a large component of overall cost, other health and social care costs also represented a significant financial burden. There were large variations in costs reported in the included studies depending on methods, country of origin, and healthcare system, but findings suggest that there are opportunities to reduce the health and economic burden related to hip fractures.

Our team recently completed a randomised controlled trial that compared a pre-discharge home assessment visit by an occupational therapist to hospital-based consultations for patients recovering from hip fracture and preparing to transition back into the community.^7^ All 77 participants in the study received hospital-based rehabilitation and discharge planning with the intervention group also receiving a home assessment visit by an occupational therapist before discharge from hospital. Primary outcomes were falls and hospital re-admissions and secondary outcomes were functional independence, health-related quality of life, and concern about falling. The primary results of this trial showed that patients who participated in a pre-discharge home assessment visit had fewer re-admissions to hospital at 30 days and 6 months, improved functional independence at 6 months, and may have had reduced falls at 30 days. There were no other between-group differences. Given that the inherent cost to health services of providing a pre-discharge home assessment visit is higher than that of providing hospital-based consultations, an economic evaluation was completed alongside the randomised controlled trial. The study aimed to evaluate the cost-effectiveness from the health service perspective of occupational therapist-led home assessment visits compared to hospital-based consultations for people recovering from hip fracture and transitioning back into the community with respect to function, falls, and health-related quality of life.

## Methods

An economic evaluation was carried out alongside an assessor-blinded, randomised controlled trial conducted between May 2016 and August 2017 in Melbourne, Australia. The trial was registered prospectively with the Australia New Zealand Clinical Trials Registry (ACTRN12616000323426) and received ethical approval from the health service and university Human Research Ethics Committees. The economic evaluation has been reported in accordance with the Consolidated Health Economic Evaluation Reporting Standards Checklist.^8^ The randomised controlled trial methods and results have been reported previously.^7,9^

Participants were eligible if they were admitted to a public hospital within the region with a primary diagnosis of hip fracture,^10^ were aged 50 years or older, were living in a private residence before their hip fracture, and had a discharge plan with an expected return to a private residence. Using a randomisation sequence with permuted blocks, participants were randomly assigned using a one-to-one allocation ratio to the intervention or the usual care group. Assignments were prepared and concealed in sequentially numbered, sealed opaque envelopes by a member of the research team not involved in recruitment or data collection. Immediately after enrolment in the trial and completion of the baseline assessment, an assignment was made by opening the next envelope in the sequence. Sample size estimation was calculated based on the clinical outcome of falls identified in the results of a previous meta-analysis^11^ and identified that a sample size of 74 participants (37 in each group) was required.

All participants received usual care after hip fracture which included assessment and management by a multidisciplinary team in hospital. For all participants, usual care involved the occupational therapist completing a standardised initial assessment, facilitating practice of daily living skills in the hospital environment, arranging assistive technology, and making referrals to home modifications services, community support services and community-based rehabilitation. Where possible, carers were involved throughout the assessment and discharge planning process. Participants allocated to the intervention group received a single home assessment visit in addition to usual care, in which the occupational therapist drove the participant to their home for a visit before hospital discharge, this typically occurred in the inpatient rehabilitation setting but could occasionally take place in the acute setting if the participant was deemed well enough to be discharged directly home. During the visit, the occupational therapist assessed the patient's needs and recorded potential hazards of the physical environment on a standardised report form. The occupational therapist facilitated practice of daily living skills at home and provided education, advice, and recommendations on assistive technology, home and task modifications, and community support services. After the home assessment visit, the patient returned to the hospital with the occupational therapist. Full details of usual care and the intervention have been published previously.^7^

The clinical outcome measures were functional independence, falls, and health-related quality of life. Outcome measures were completed at 30 days and 6 months post-discharge. Functional independence was measured using the Functional Independence Measure (FIM),^12^ where a 22-point change in score indicates the minimal clinically important difference. Falls were defined as unexpected events where participants came to rest on the ground, floor, or lower level.^13^ Falls were monitored by participants recording incidents on a monthly self-report calendar. Health-related quality of life was captured through a multi-attribute utility instrument, the EQ-5D-3L which is responsive to change for patients following hip fracture.^14^ The EQ-5D was converted into a utility index (scale of 0–1) to report quality-adjusted life years.^15^

The economic evaluation was conducted from a health service perspective to align with the inpatient hospital study setting. The time horizon for the costs included in the primary analysis commenced with the acute hospital admission post hip fracture and included any subsequent transfer for a rehabilitation admission as well as any hospital re-admissions within 30 days of discharge. The pre-discharge home visit intervention occurred during the acute or rehabilitation hospital admission. As the intervention was a pre-discharge home assessment during an inpatient hospital stay, aligning costs to the inpatient admission and any immediate re-admissions ensures direct health service costs are captured. In addition to 30 days, the secondary endpoint for effect was at 6 months post-discharge and included any hospital re-admissions within that period, to examine the medium-term impact of the intervention. All available data were used for analysis without using imputation methods.

The dates for the resource utilisation and subsequent costs commenced in May 2016 and concluded in June 2017. All costs were presented in Australian dollars. Admissions that were costed in the 2015–2016 financial year were inflated by 1.9% (National Consumer Price Index^16^) to enable all cost data to be presented in 2016–2017.

Health service utilisation and costs were gathered from two data sources. Utilisation data, including acute and rehabilitation admissions, any re-admissions within 30 days post-discharge, and occupational therapy session details in minutes, was collected from medical records using a purpose-built data collection form. Cost data for hospital admissions and re-admissions within 30 days were collected from the health service administration database. Cost data for the intervention were modelled based on the average time taken for a pre-discharge home visit by participating occupational therapists. Redistribution of occupational therapy costs was only necessary for this visit, as both usual care and intervention groups received similar services otherwise.^7^ The cost was calculated by multiplying the average time for the visit (including the home visit, travel time, and report writing) by the wage rate of a mid-level occupational therapist (grade 2 year 2) according to the Health Service employment agreement,^17^ inflated by the consumer price index^16^ to achieve a 2016–2017 wage rate, then further inflated by 25% for on-costs ($46.35/hour), as well as the cost of travel based on 65 cents per kilometre.^18^ With a time horizon of less than 1 year (acute admission to 6-month follow up), no discounts were applied. Opportunity costs were not included in this economic evaluation as they did not alter the acute or rehabilitation admission costs; that is, opportunity costs were absorbed within usual duties of the staff. In addition, acute and rehabilitation costs were combined, regardless of whether the home visit took place in an acute or rehabilitation setting, as this study considered that the episode of care began on admission to acute and concluded on return to the community.

### Cost-effectiveness analysis

The economic evaluation included a cost-effectiveness analysis comparing the direct health service costs between usual care and the intervention group, covering acute hospital admission after hip fracture, immediate rehabilitation transfers, and any hospital re-admissions within 30 days of discharge. The primary outcome measure was cost-effectiveness of pre-discharge home visits per minimal clinically important difference (22 points) on the FIM. Secondary outcome measures were cost-effectiveness of pre-discharge home visits per change in rate of falls and change in quality-adjusted life years using utility index data at 30 days and 6 months. To complete a cost-effectiveness or cost-utility analysis there needs to be a difference in both cost and effect. The difference may be statistically significant, or it may represent a trend toward a difference. If no difference in cost and/or effect exists, the cost-effectiveness analysis becomes redundant as either the numerator or the denominator represents zero. In this study planned cost-effectiveness and cost-utility analyses were only completed when there was a difference or at least a trend towards a difference in both cost and effect.

Cost-effectiveness and cost-utility data were presented in the form of an incremental cost-effectiveness ratio, which represents the cost-per-unit of the effect of an intervention. The incremental cost-effectiveness ratios were calculated by dividing the incremental cost of an intervention by the incremental effect of the intervention. The incremental cost-effectiveness ratio was calculated as follows: Incremental cost-effectiveness ratio = average costs of intervention – average costs of usual care/average effects of intervention – average effects of usual care. This approach was represented graphically on a cost-effectiveness plane, with the x-axis showing the effectiveness domain and the y-axis showing the cost domain. The most favourable interventions fall in the quadrant with lower costs and higher effectiveness (dominant quadrant), while the least desirable are in the quadrant with higher costs and lower effectiveness (North-West quadrant). The uncertainty of the estimates was calculated using the bootstrapping method with 5000 repetitions.^19^ For the cost-effectiveness and cost-utility analyses, the incremental cost-effectiveness ratio was reported with a standard deviation and 95% confidence intervals (CIs) as well as presented as a point estimate with 50%, 75%, and 95% confidence ellipses. Significance was reported as *p* < 0.05 and SPSS^20^ and a purpose-built Excel database^19^ were used for the analyses. Details of the analysis of the effect of the intervention are described elsewhere.^7^

## Results

There were 77 participants randomised to either the usual care group (hospital-based occupational therapy assessment, *n* = 40) or the intervention group (usual care and pre-discharge home assessment visit, *n* = 37). By 6 months, nine participants had died (intervention *n* = 5, usual care *n* = 4). Seven participants did not receive the intervention as allocated (intervention: *n* = 3 discharged to residential care, *n* = 1 declined visit; usual care: *n* = 1 died, *n* = 1 withdrew, *n* = 1 received home visit). Baseline characteristics appeared well-matched in both groups (Table 1).^7^ The study population was predominantly female (*n* = 55/77, 71%) and had a mean age of 82.2 (standard deviation (SD) 7.3) years. Before their hip fracture the majority walked independently with or without a stick (*n* = 63/77, 82%) and were independent completing personal care tasks (*n* = 66/77, 86%). Majority of participants were discharged from inpatient rehabilitation (*n* = 74/77, 96%). Both the usual care and intervention groups received a similar number of occupational therapy sessions, except for the home assessment visit (intervention group: 8.0 sessions, SD 4.0; usual care: 7.1 sessions, SD 3.0).^7^ On average, providing the intervention required 142 minutes, which included the home visit, travel time, and report writing, as well as an average distance of 20 km (40 km round trip), travelled per home visit. Based on a cost of $46.35 per hour for clinician time, as well as 65c per km travelled, this was an intervention cost of $135.70 per home visit completed.

**Table 1.** Participant characteristics.

Participant characteristics	Intervention (*n* = 37)	Control (*n* = 39)^#^
Age *(years)*, mean (SD)	83.4 (7.1)	80.9 (7.3)
Gender, n females (%)	28 (76)	27 (68)
Co-morbidities, n mean (SD)	5.2 (2.4)	5.2 (2.8)
Living situation, n (%) Alone With others	13 (35) 24 (65)	18 (46) 21 (54)
Support, n (%) None Family Home care	17 (46) 8 (22) 12 (32)	18 (46) 8 (21) 13 (33)
Falls history, n yes (%)	12 (32)	7 (18)
Pre-morbid mobility, n (%) Independent/SPS Frame/ wheelchair	29 (78) 8 (22)	34 (87) 5 (13)
Pre-morbid personal care, n (%) Independent Assistance	33 (89) 4 (11)	33 (85) 6 (15)
History of cognitive decline, n yes (%)	1 (3)	7 (18)
Discharging hospital Acute Inpatient rehabilitation OT occasions of service, mean (SD) Discharge destination Home Residential care Died in hospital Community-based rehabilitation* OT PT Total	1 (3) 36 (97) 8.0 (4.0) 34 (92) 3 (8) 0 (0) 7 11 18/34	2 (5) 37 (95) 7.1 (3.0) 34 (87) 4 (10) 1 (3) 1 12 13/35

*Available after discharge for patients assessed as requiring further rehabilitation services.

# One participant withdrew.

*n*: Number; OT: occupational therapy; PT: physiotherapy; SD: standard deviation; SPS: single point stick.

Details of the clinical outcomes have been reported in detail elsewhere.^7^ Briefly, there was no difference in function at 30 days but patients in the intervention group had a significantly higher level of function at 6 months than those in the usual care group (mean difference 11.2 units 95% CI 4.2 to 18.2). The rate of falls at 30 days and 6 months was not significantly different between groups, but the results favoured the intervention group at 30 days as there were fewer falls in this group (*n* = 6 falls from 6 participants) compared with the usual care group (*n* = 14 falls from 11 participants) with an incidence rate ratio of 0.41 (95% CI 0.15 to 1.11). At 6 months, there was no difference in the rate of falls between the intervention and usual care group (incidence rate ratio 0.91, 95% CI 0.39 to 2.09). There was no between-group difference in health-related quality of life at 30 days (mean difference 0 units, 95% CI −0.1 to 0.1) or at 6 months (mean difference 0 units, 95% CI −0.1 to 0.2). There were a small number of data points greater than two standard deviations above the mean for the primary continuous outcomes, representing participants who transferred to residential aged care or were receiving a high level of support from family at home. As these data represented a natural part of our population, we did not complete sensitivity analyses.

Total health service cost per patient was $38,157 (SD $17,745) for the intervention group and $44,389 (SD $34,803) for the usual care group, a non-significant between-group difference of $6182 (95% CI −$6414 to 18,777, *p *= 0.33) favouring the intervention group. The cost was broken down into acute, rehabilitation, and 30-day re-admission categories with each result suggestive of a lower cost for the intervention group (Table 2). It is noted that in the rehabilitation phase, the usual care group had a shorter length of stay, yet a higher cost, most likely due to higher pathology and imaging costs.

**Table 2.** Cost and health service utilisation data.

	Intervention (home assessment) group (*n* = 37)	Usual care group (*n* = 39)	Mean difference (95% CI) (usual care minus control)	Significance
Total LOS, days (SD)	32.9 (21.6)	31.6 (20.9)	−5.0 (−18.3 to 8.3)	*p *= 0.46
Total cost, dollars* (SD)	$38,157 ($17,745)	$44,389 ($34,803)	$6182 (−$6414 to $18,777)	*p *= 0.33
Acute LOS, days (SD)	7.8 (5.0)	9.3 (11.1)	1.4 (−2.5 to 5.4)	*p *= 0.46
Acute cost, dollars* (SD)	$22,734 (14,878)	$23,455 ($29,544)	$721 (−$9945 to $11,386)	*p *= 0.89
Rehabilitation LOS, days (SD)	25.1 (19.1)	22.3 (15.9)	−2.8 (−11.7 to 6.2)	*p *= 0.54
Rehabilitation cost, dollars* (SD)	$14,199 ($14,174)	$17,601 ($21,477)	$3403 (−$4891 to $11,696)	*p *= 0.42
30-Day re-admission LOS, days (SD)	0.03 (0.16)	1.64 (3.99)	1.61 (0.32 to 2.90)	*p *= 0.16
30-day re-admission cost, dollars* (SD)	$1224 ($6295)	$3283 ($12,753)	$2059 (−$2527 to $6644)	*p *= 0.37

*All costs presented in Australian dollars.

CI: confidence interval; *n*: number; LOS: length of stay.

No cost-effectiveness analyses were completed for the functional change score at 30 days, the rate of falls at 6 months, or the change in quality-adjusted life years at both 30 days and 6 months as no difference in effect was detected for these outcomes.

**Figure 1.**
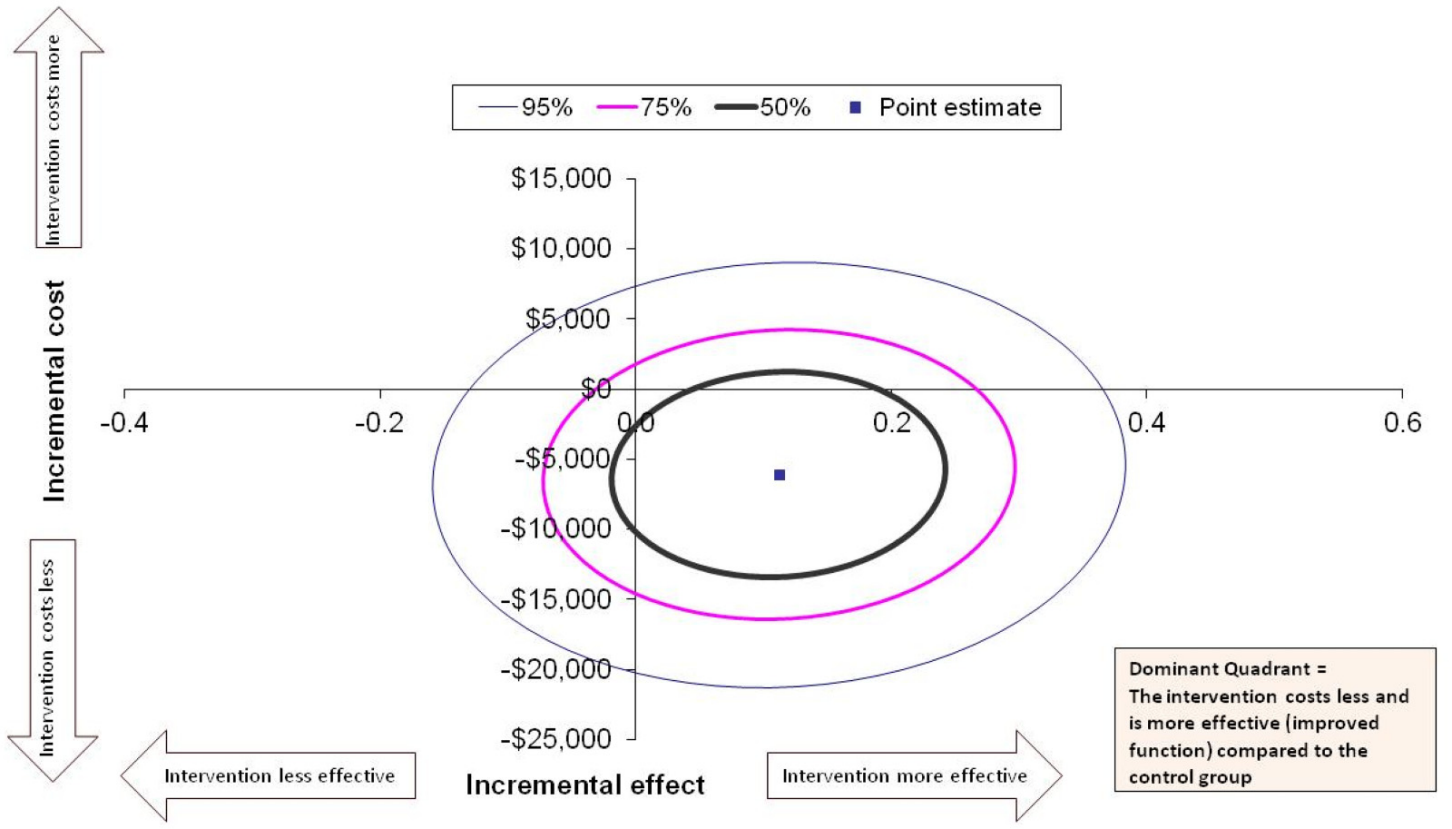
Cost-effectiveness for achieving a Minimal Clinically Important Difference (MCID) in Functional Independence Measure (FIM) at 6 months. All costs presented in Australian dollars.

The incremental cost-effectiveness ratio for achieving a minimal clinically important difference in FIM score at 6 months was a cost saving of $71,337 (SD $499,243; 95% CI to −$998,930 to $411,409) per additional minimal clinically important difference in FIM achieved in favour of the intervention group. Figure 1 displays the point estimate in the dominant quadrant (South-East quadrant when a higher effect score is favourable) to indicate that the cost was less and the rate of achieving a minimal clinically important difference in FIM was higher for the intervention group compared to usual care. The majority of the 50%, 75%, and 95% confidence ellipses were also in the dominant quadrant.

**Figure 2.**
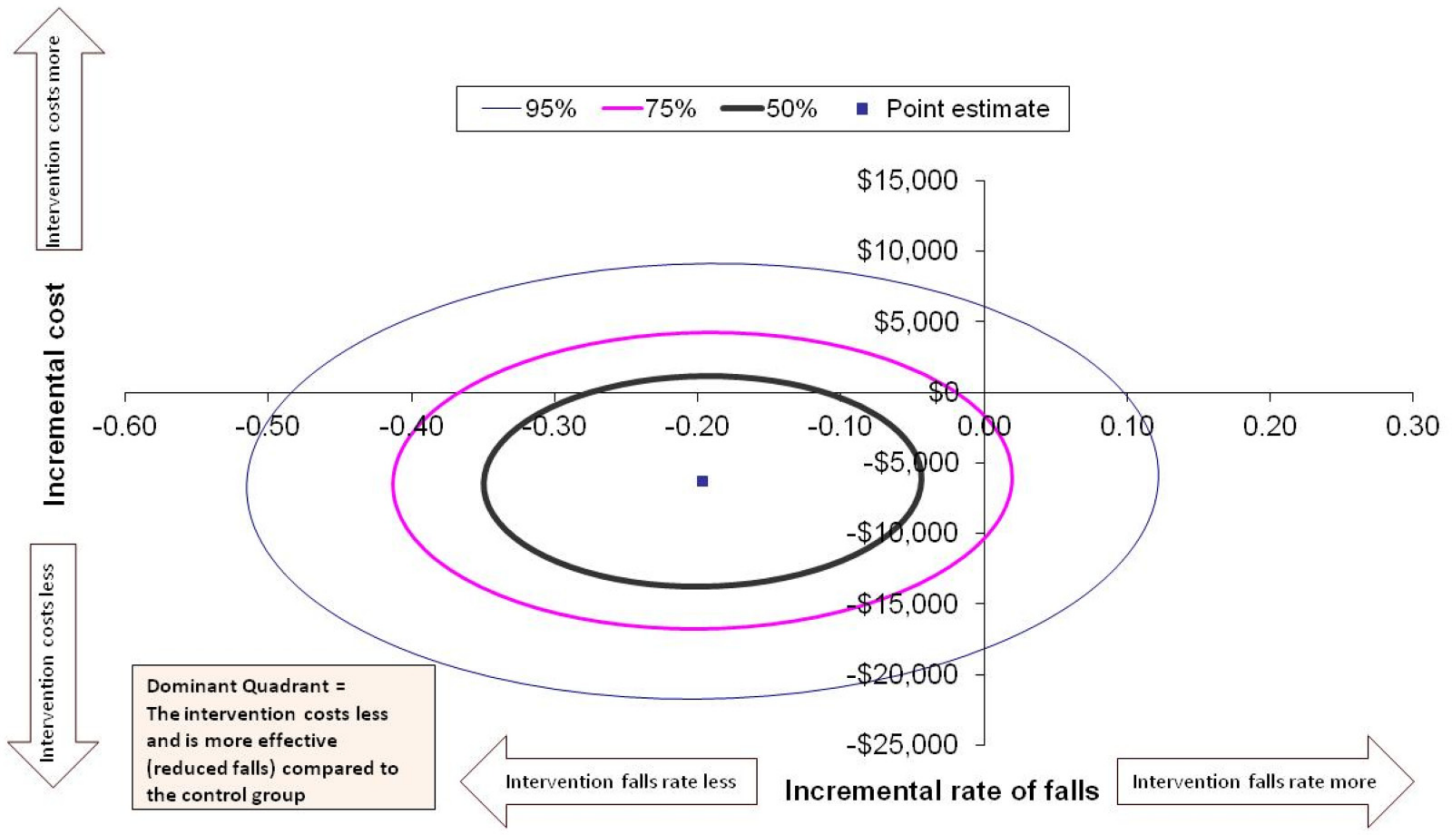
Cost-effectiveness for the rate of falls at 30 days. All costs presented in Australian dollars.

The incremental cost-effectiveness ratio for the rate of falls at 30 days was a cost saving of $34,832 (SD $189,076; 95% CI −$331,344 to $213,900) per fall avoided in favour of the intervention group. Figure 2 displays the point estimate in the dominant quadrant (South-West quadrant when a lesser effect score is favourable) to indicate that the cost and rate of falls are less for the intervention group compared to usual care. The majority of the 50%, 75%, and 95% confidence ellipses are also in the dominant quadrant.

## Discussion

From a health service perspective, pre-discharge home assessment visits for patients after hip fracture are likely to be cost-effective in relation to restoring functional independence and reducing falls, but not with respect to quality of life. Our results show that compared to usual care, participants who received a pre-discharge home assessment visit likely had fewer falls in the 30 days after discharge, increased functional independence at 6 months, and a reduction in cost to the health service. The confidence interval ellipses for the incremental cost-effectiveness ratios show that most of the confidence intervals fell within the dominant quadrant, suggesting that the intervention is likely to be dominant over the comparator of usual care. Compared to three similar studies that incorporated an economic evaluation of home environmental assessments,^21–23^ our results may have been more favourable as the intervention was focused on patients recovering from hip fracture who are at high risk of falls.

The intervention was likely cost-effective by reducing the risk of falls in this high-risk population, with lower hospital re-admission costs in the home assessment group outweighing the higher occupational therapy staff and travel costs associated with the intervention. Hospital re-admission is a problem because it affects the health and well-being of patients and represents a financial burden to the health system. Consistent with previous research, our results indicate that providing support in the transition from hospital to the home environment can reduce the risk of re-admission.^24–26^ The intervention provided within our study follows strategies^27^ that lead to a reduction in hospital re-admissions while minimising the financial investment, including, (1) matching the intensity of the intervention to the patient's risk of re-admission, (2) using interventions with a lasting effect and (3) targeting high-risk groups. In addition, a systematic review of economic evaluations of interventions designed to reduce re-admissions identified that interventions that engaged patients and caregivers offered the greatest value to the health system.^28^ In our study, an occupational therapist worked with patients in a high-risk group and their families to provide recommendations and introduce additional support where required providing them with the best opportunity to maximise their ability to remain living independently in the community.

Home assessment visits for high-risk patients have the potential to improve care and reduce re-admissions without increasing overall hospital spending, but limited resources may restrict their implementation. A recent audit of current practice showed that only 20% of patients after hip fracture participated in a home visit.^29^ Our results provide support for the contention that providing more occupational therapists to complete pre-discharge home assessments may be a cost-effective use of resources. Another approach may be the use of telerehabilitation, which has been suggested as an effective and viable way to access home modification services.^8^ Any opportunity to view and discuss the home environment with an occupational therapist enables the older person to discuss alternative ways to solve problems based on their unique knowledge of their roles and routines, resulting in individually tailored strategies to enhance independence.^27^

The strengths of this economic evaluation are that it was completed alongside a randomised controlled trial, and it was reported according to the Consolidated Health Economic Evaluation Reporting Standards (CHEERS) checklist.^8^ The study did have some limitations. The sample size for the cost-effectiveness analysis was based on the clinical outcome of falls so a clinically based sample size may not provide adequate power for economic evaluation. However, given that an economic evaluation is not typically concerned with hypothesis testing but is more about estimation, it can still provide useful information. Only re-admissions to public hospital facilities within the region were included. Some re-admissions may have occurred to private facilities or outside the region, but there is no reason to believe that the distribution of any missed re-admissions was likely to differ between groups. Cost data was from several years ago, which may not fully reflect current clinical practice. However, it still offers valuable insights into the effectiveness of the intervention and serves as a useful guide. Cost data were modelled on the average time taken to complete a pre-discharge home assessment visit rather than individual patient data. The follow-up period of the study provided the medium-term impact of the intervention and so further research is required to understand the long-term impact. The economic outcome of home visits for patients with hip fractures from a societal perspective was beyond the scope of this study and as this is a limitation, it warrants further research. There may be some limitations to generalisability to health systems in other countries where requirements for insurance, limitations related to geography and lack of transport may limit the feasibility of interventions with patients outside hospital settings.

From a health service perspective, for patients recovering after hip fracture pre-discharge home assessment visits by an occupational therapist are likely to be cost-effective in restoring functional independence and reducing falls.

**Clinical messages**

- From a health service perspective, pre-discharge home assessment visits are likely to be cost-effective in restoring functional independence and reducing falls in patients after hip fracture.
- The likely cost-effectiveness of pre-discharge home assessment visits for patients recovering after hip fracture adds to the previous evidence that they reduce re-admissions to hospital, increase functional independence, and may reduce falls.
- Involvement of occupational therapists in the multidisciplinary team to facilitate discharge planning after hip fracture is likely cost-effective.

## Supplemental Material

sj-docx-1-cre-10.1177_02692155241312065 - Supplemental material for An economic evaluation of pre-discharge home assessment visits following hip fracture: Analysis from a randomised controlled trial Supplemental material, sj-docx-1-cre-10.1177_02692155241312065 for An economic evaluation of pre-discharge home assessment visits following hip fracture: Analysis from a randomised controlled trial by Kylee J. Lockwood, Nicholas F. Taylor, Katherine E. Harding and Natasha K. Brusco in Clinical Rehabilitation
